# High-efficiency non-diffractive generator of arbitrary vectorial optical fields with minimal optical elements

**DOI:** 10.1016/j.optcom.2020.125443

**Published:** 2020-05-15

**Authors:** Billy Lam, Chunlei Guo

**Affiliations:** The Institute of Optics, University of Rochester, Rochester, NY, 14627, USA

**Keywords:** Spatial light modulator, Beam shaping, Optical fields

## Abstract

Tailoring electromagnetic waves has a wide range of applications, such as optical trapping, focus engineering, imaging, laser cutting, and optical communication. To do so, the spatial distribution of at least one of the four degrees of freedom of electromagnetic waves, amplitude, phase, polarization ratio, and retardance, must be modified. Arbitrary vectorial optical fields (VOF) can be engineered by spatially modulating all four degrees of freedom simultaneously. However, existing dynamic vectorial optical field generators (VOF-Gens) require intensive alignment and many optical elements in order to achieve high efficiency. Here, we design a dynamic VOF-Gen that can generate arbitrary VOFs using only five optical elements. Experimentally, we demonstrated an efficiency of 72%, the highest ever demonstrated.

## 1 Introduction

The shaping of optical fields has various practical applications in optical trapping, focus engineering, laser surface structuring, imaging, laser cutting, optical communication, and many more [1], [2], [3], [4], [5], [6], [7], [8], [9], [10], [11]. Optical trapping enables researchers to manipulate particles for studying biophysics, chemistry, and biology [1], [2]. Beating the diffraction limit is one of the most important aspects of optical imaging, microscope systems, laser cutting, and nanoscale fabrication [3], [4]. In addition, light beam focus engineering allows the patterning of a wide array of designs for laser surface structuring [5], [6], [7]. Optical communication utilizes polarization and orbital angular momentum of light as information carriers for free space communication [8]. By shaping optical fields, one can obtain a range of highly desirable types of beams, such as flat top, Bessel beam, vortex beams, and radially and azimuthally polarized beams. The flat top is useful in a wide range of applications such as material processing and lithography. Bessel beam has been demonstrated for optical trapping, rapid three-dimensional imaging of living cell, and material processing [1], [9], [12], [13]. Vortex beam, radially and azimuthally polarized beams are used in material processing and optical trapping [14], [15], [16].

Tailoring electromagnetic waves involves controlling the spatial distribution of the four degrees of freedom of electromagnetic waves, i.e. amplitude, phase, polarization ratio, and retardance. Generation of vectorial optical fields, which has spatially inhomogeneous states of polarization, can be realized statically or dynamically [5], [17], [18], [19], [20], [21], [22], [23], [24], [25], [26], [27], [28], [29], [30], [31]. Many static vectorial optical field generators (VOF-Gens) is achieved by a single optical element, such as a spatial-variant wave plate [17], a subwavelength grating [18], and a metasurface [19], [20]. Single element VOF-Gens can achieve very high efficiency beyond 90% as they are based on the birefringence effect. Other static vectorial optical field generators (VOF-Gens) includes Michelson [21] and Sagnac [22] interferometric techniques that generate cylindrical VOF by superimposing two beams with opposite orbital angular momentums, which are generated by having an odd number of reflections in one interferometer arm and even number of reflections in the other. Both configurations achieve high efficiency up to 87% [21], [22].

Dynamic VOF-Gens involves spatial light modulator (SLM) or digital micromirror device (DMD), allowing multiple degrees of freedom of electromagnetic waves to be spatially manipulated dynamically [5], [23], [24], [25], [26], [27], [28], [29], [30], [31]. Hence, dynamic VOF-Gens are more flexible. However, current dynamic VOF-Gens are either inefficient and/or require many optical elements with intensive alignment [23], [24], [25], [26], [27], [28], [29]. There are two types of dynamic VOF-Gen, the diffractive type and the non-diffractive type. Currently, the most efficient dynamic VOF-Gen is the diffractive type that utilizes the superposition of orthogonal polarizations with an efficiency of about 60% [32]. In a diffractive type dynamic VOF-Gens, a superpixel consisting of at least 2 × 2 subpixels diffracts a beam into multiple orders with controllable complex amplitudes that recombine coaxially after converting into orthogonal polarizations via waveplates or polarizers [25], [26], [27], [28], [29], [32]. These VOF-Gens can be realized by splitting the beams in various ways, such as using polarizer [27], [28], Wollaston prism [26], computer-generated-hologram [25], or binary-amplitude-computer-generated-hologram [29]. Many configurations of the diffractive type dynamic VOF-Gen require only one SLM by splitting the SLM panel into two sections to modulate both orthogonal polarizations independently. However, all these setups require intensive alignment and many optical elements. In addition, splitting the beam into different paths and recombining can degrade the beam quality and stability. Therefore, these methods are intrinsically less stable than those that do not rely on superposition.

Non-diffractive based dynamic VOF-Gens that do not rely on superposition are very stable and robust. The tradeoff is the complexity of VOF-Gen as the beam requires passing through SLM panels four times to achieve independent control on phase, amplitude, and polarization distribution. One example is Zhan’s dynamic VOF-Gen that double passes two reflective SLM in four sections to generate arbitrary vector fields [24]. This dynamic VOF-Gen is extremely inefficient because the beam double passes four non-polarizing beam splitter, significantly reducing the output power to less than 1∕256 of the input power. Furthermore, their setup requires intensive alignment as there are three 4f-systems to relay the optical field from one reflective-SLM panel to the next. In order to achieve high efficiency, SLM must be used at a non-zero incident angle or in transmission mode [31]. Kenny et al. adopted this strategy to modulate only three out of the four degrees of freedom of electromagnetic waves with a theoretical efficiency of 100% [31]. The approach of using SLM at a non-zero incident angle, however, compromises the performance of the SLM, forbids a perfect relay of the electric field in a 4f-system and increases the difficulty of alignment. A highly-efficient compact dynamic VOF-Gen without the shortcoming of superimposing orthogonal polarizations and using SLMs at non-zero incident angles is currently lacking.

The low efficiency and/or intensive alignment of dynamic VOF-Gen with many optical elements has thwarted its many practical aforementioned applications. Here, we design a non-diffractive-based dynamic VOF-Gen that has 100% theoretical efficiency with only five optical elements. Hence, the setup is very simple and compact. Moreover, the setup is robust and stable as it does not rely on superposition where a beam is split and recombined. By making the dynamic VOF-Gen energy efficient, stable and reducing the number of optical elements down to five, we bring the dynamic VOF-Gen significantly closer to practicality for all kinds of applications in the fields of biophysics, chemistry, optics and many more.

## 2 Method

In order to modulate all four degrees of freedom of electromagnetic waves, a minimum of four wave plates and a polarizer is required for non-diffraction based dynamic VOF-Gen. Thus, four panels of SLM are required. because the effect of wave plates is dependent on the incident polarization, the four modulations will be phase, amplitude, polarization ratio, and retardance in that order.

Mathematically, the incident beam for the dynamic VOF-Gen is assumed to be monochromatic because the SLMs are not achromatic. For our derivation, the incident beam is assumed to be horizontally polarized for our particular design. The incident beam undergoes modulation as it propagates through the SLM. The modulation can be described mathematically using the Jones matrix. Since the SLM with crystal axis at some angle θ is a spatial distribution of voltage-controlled wave plate, individual SLM pixel can be described as

(1) $$M\left(\phi ,\theta \right)=R\left(\theta \right)\left(\begin{array}{cc} \text{exp}\left(i\phi \right) & 0\\ 0 & 1 \end{array}\right)R\left(-\theta \right)$$

where ϕ is the retardance, and R(θ) is the rotation matrix:

(2) $$R\left(\theta \right)=\left(\begin{array}{cc} \cos\theta & -\sin\theta \\ \sin\theta & \cos\theta \end{array}\right).$$

The full expression is

(3) $$M\left(\phi ,\theta \right)=\left(\begin{array}{cc} \text{exp}\left(i\phi \right)\cos^{2}\theta +\sin^{2}\theta & \left(\text{exp}\left(i\phi \right)-1\right)\sin\theta \cos\theta \\ \left(\text{exp}\left(i\phi \right)-1\right)\sin\theta \cos\theta & \text{exp}\left(i\phi \right)\sin^{2}\theta +\cos^{2}\theta \end{array}\right)$$

**Fig. 1:**
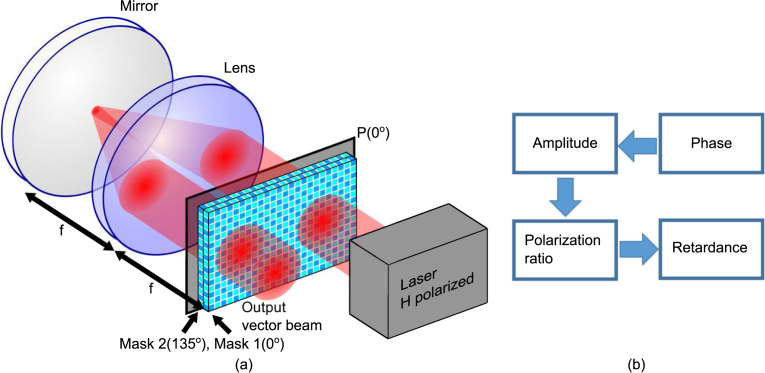
(a) Schematic diagram of the proposed dynamic VOF-Gen. (b) Flow chart of the modulation. P - Polarizer.

Fig. 1(a) shows the schematic diagram of the setup. The setup consists of a two-dimensional dual-mask transmissive-SLM (TSLM) and a reflective 4f-system. The incident beam passes through the right half of the TSLM panels. The optical field is then relayed to the left half of the TSLM by a reflective 4f-system, double passing the polarizer and TSLM. Unless the polarizer is ultrathin, it must cover the path of the back-reflected light as well to maintain the distance of f from the lens to the SLM panel in the 4f system. The polarizer selects the horizontal polarization in both passes. We separate the SLM modulations into two sections. The first section is the phase and amplitude modulation during the first pass of the TSLM while the second section is polarization ratio and retardance modulation during the second pass of the TSLM. This sequence of modulation is depicted in Fig. 1(b).

### 2.1 Phase and amplitude modulation

The first section has two panels where the first panel has a crystal axis along $\theta_{1}=0^{\circ }$ (horizontal) and the second panel has a crystal axis along $\theta_{2}=135^{\circ }$. A pixel of the first panel can be described as

(4) $$M\left(\phi_{1},0^{\circ }\right)=\left(\begin{array}{cc} \text{exp}\left(i\phi_{1}\right) & 0\\ 0 & 1 \end{array}\right).$$

A pixel of the second panel can be written as

(5) $$M\left(\phi_{2},135^{\circ }\right)=\frac{1}{2}\left(\begin{array}{cc} 1+\text{exp}\left(i\phi_{2}\right) & 1-\text{exp}\left(i\phi_{2}\right)\\ 1-\text{exp}\left(i\phi_{2}\right) & 1+\text{exp}\left(i\phi_{2}\right) \end{array}\right).$$

A horizontally polarized beam, $U_{1}\left(x,y\right)={\left(U_{1}\left(x,y\right)0\right)}^{T}$ where T denotes transpose, passing through the first two panels followed by a linear polarizer at 0° will result in a field described by

(6) $$U_{2}^{\prime }\left(x,y\right)=\left(\begin{array}{cc} 1 & 0\\ 0 & 0 \end{array}\right)M\left(\phi_{2},135^{\circ }\right)M\left(\phi_{1},0^{\circ }\right)\left(\begin{array}{c} U_{1}\left(x,y\right)\\ 0 \end{array}\right)$$

(7) $$=U_{1}\left(x,y\right)\text{exp}\left(i\left(\phi_{1}+0.5\phi_{2}\right)\right)\left(\begin{array}{c} \cos\left(\frac{\phi_{2}}{2}\right)\\ 0 \end{array}\right)$$

Upon exiting the linear polarizer, the amplitude and phase of the field are modulated. The amplitude of the output beam is attenuated by a factor of $\cos\left(\phi_{2}∕2\right)$. The phase is increased by an amount of $\phi_{1}+0.5\phi_{2}$. Because these two quantities are linearly independent, the phase and amplitude can be modulated independently.

### 2.2 Polarization modulation

After the amplitude and phase modulation, the field is relayed to the second SLM section by a reflective 4f-system. Because the optical fields from the Fourier plane to the focus are related by a Fourier transform, F, the optical field at the third SLM panel is

(9) $$U_{3}\left(x,y\right)=F\left\{\left(\begin{array}{cc} 1 & 0\\ 0 & -1 \end{array}\right)F\left\{U_{2}^{\prime }\left(x,y\right)\right\}\right\}=U_{2}^{\prime }\left(-x,-y\right)$$

The reflective 4f-system is important so that the field is relayed from the second panel of the SLM to the third panel without any diffraction. Both Cartesian coordinates are flipped after the two Fourier transform operations. On the other hand, a 4f-system is not necessary from the 1st to the 2nd or 3rd to the 4th because the two masks in the TSLM are separated by only a few microns so there is hardly any diffraction effect. In reality, the diffraction effect from the misalignment of the reflective 4f-system should be the primary concern.

This returning field double passes through the SLM but at a new position. This allows the field to be modulated again; this time is a polarization modulation. This section also has two panels, the third panel has a crystal axis at 45° and the fourth panel has a crystal axis at 0°. The reason why the third crystal axis is 45° is that the reflected beam sees the mirrored angle. A pixel of the third panel can be written as

(10) $$M\left(\phi_{3},45^{\circ }\right)=\frac{1}{2}\left(\begin{array}{cc} 1+\text{exp}\left(i\phi_{3}\right) & -1+\text{exp}\left(i\phi_{3}\right)\\ -1+\text{exp}\left(i\phi_{3}\right) & 1+\text{exp}\left(i\phi_{3}\right) \end{array}\right)$$

A pixel of the fourth panel is described by

(11) $$M\left(\phi_{4},0\right)=\left(\begin{array}{cc} \text{exp}\left(i\phi_{4}\right) & 0\\ 0 & 1 \end{array}\right).$$

Upon passing through the third and fourth SLM panels, the output beam becomes

(13) $$U_{4}^{\prime }\left(x,y\right)=M\left(\phi_{4},0^{\circ }\right)M\left(\phi_{3},45^{\circ }\right)U_{2}^{\prime }=U_{1}\left(-x,-y\right)\text{exp}\left(i\left(\phi_{1}+0.5\phi_{2}+0.5\phi_{3}+\phi_{4}\right)\right)\times \cos\left(\frac{\phi_{2}}{2}\right)\left(\begin{array}{c} \cos\left(\frac{\phi_{3}}{2}\right)\\ \sin\left(\frac{\phi_{3}}{2}\right)\text{exp}\left(-i\left(\phi_{4}-\frac{\pi }{2}\right)\right) \end{array}\right)$$

The detailed derivation of the above equation can be found in the supplementary material. It can be seen that the polarization ratio depends solely on $\phi_{3}$ and the retardance depends only on $\phi_{4}$. By controlling polarization ratio and retardance independently, arbitrary polarization state can be achieved. Similarly, the amplitude depends solely on $\phi_{2}$ with an attenuation factor of $\cos\left(\phi_{2}∕2\right)$. Thus, this dynamic VOF-Gen is theoretically 100% energy efficient given that the optical elements are lossless. Realistically, the main source of loss in this system, if one is built, would be from the TSLM. The phase is dependent on all $\phi_{1},\phi_{2},\phi_{3},\phi_{4}$. Arbitrary phase can be achieved by varying $\phi_{1}$ independently.

Therefore, all four degrees of freedom of the electromagnetic radiation of phase, amplitude, polarization ratio, and retardance can be controlled independently. In order to fully modulate phase, amplitude, polarization ratio, and retardance, they must be able to be tuned across all possible range. The phase has a range of 0 to 2π, the amplitude has a range from 0 to $U_{1}\left(x,y\right)$, the polarization ratio has a range of 0 to infinity, and the retardance has a range from 0 to 2π. As a result, the mask 2 of the SLM (2nd, 3rd panels) requires birefringent from 0 up to π for full modulation of amplitude and polarization ratio. Similarly, the mask 1 (1st, 4th panels) requires birefringent up to 2π for full modulation of phase and retardance. Note that the identity exp(iπ)=−1 takes place in the vertical polarization term of Eq. (13) in order to achieve all possible polarization state. However, unfortunately there is no commercially available 2D dual mask transmissive SLM with the desired configuration. Because of this, we demonstrate the simultaneous modulation of both phase and amplitude by double passing a single mask using the described concept. The same is done for polarization modulation to fully demonstrate the proof of principle of our proposed concept. The experiment and results are detailed in the next section.

## 3 Proof of principle

**Fig. 2:**
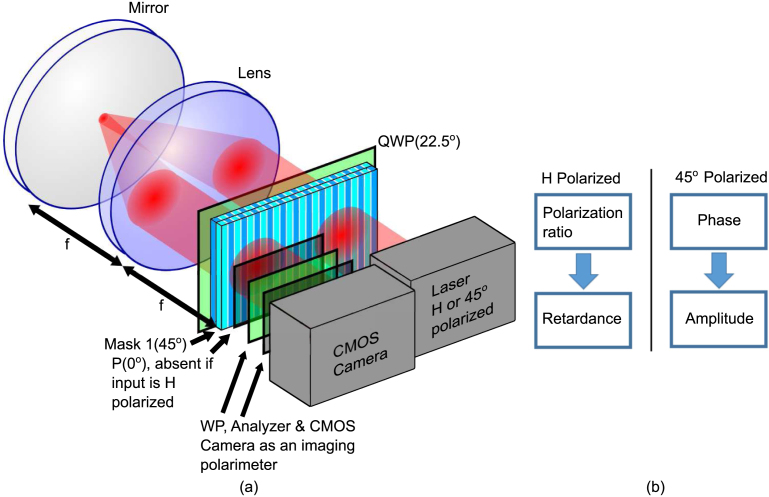
(a) Schematic diagram of the experimental setup as a proof of principle for the dynamic VOF-Gen. (b) Flow chart of the modulation for incident horizontal polarization and 45° polarization. QWP — quarter-wave plate, WP — waveplate.

As a proof of principle, we used the Cambridge Research & Instrumentation (CRi) Spatial Light Modulators with a one-dimensional array of 128 pixels and a pixel pitch of 100 microns to structure a vector field. The CRi SLM is a dual mask SLM that uses nematic liquid crystals, which provides an electrically variable index of refraction for light that is polarized along the extraordinary axis of the crystal. The first mask has the extraordinary axis at 45°. The second mask has the extraordinary axis at 135°, but it will not be used because it is not 45° with respect to the first mask. The configuration, which is similar to Fig. 1, is shown in Fig. 2. A Ti:Sapphire laser is used in continuous wave mode with central wavelength of 820nm. The beam is incident into our proof of principle device followed by an imaging polarimeter consisting of a wave plate, an analyzer and a complementary metal–oxide semiconductor (CMOS) camera. The model number of the CMOS camera is ZWO ASI 178 uncooled monochrome CMOS telescope camera. The beam is attenuated below the saturate of the camera. The imaging polarimeter measures the Stokes parameters as follows. First, HWP at 0° and 45° and polarizer at 0° are used to measure $S_{0}$ and $S_{1}$. Then, the HWP is rotated to $22.5^{\circ }$ and $77.5^{\circ }$ to measure $S_{2}$. Lastly, the HWP is replaced with a QWP rotated to 45° and 135° to measure $S_{3}$. To simulate the two crystal axis to be 45° with respect to each other, we only use the first SLM panel but rotate the polarization of the beam by 45° by double passing a quarter-wave-plate as the field return to the SLM panel again using a reflective 4f-system. The reflective 4f-system was aligned by checking the collimation of the output using a shearing interferometer [33]. When the input beam is horizontally polarized, the setup modulates the polarization because the SLM crystal axis is 45° with respect to the input polarization. When the input beam is 45° polarized, the setup modulates the phase and amplitude because the SLM crystal axis is the same as the input polarization. The sequence of modulation is shown in the flow chart of Fig. 2(b).

**Fig. 3:**
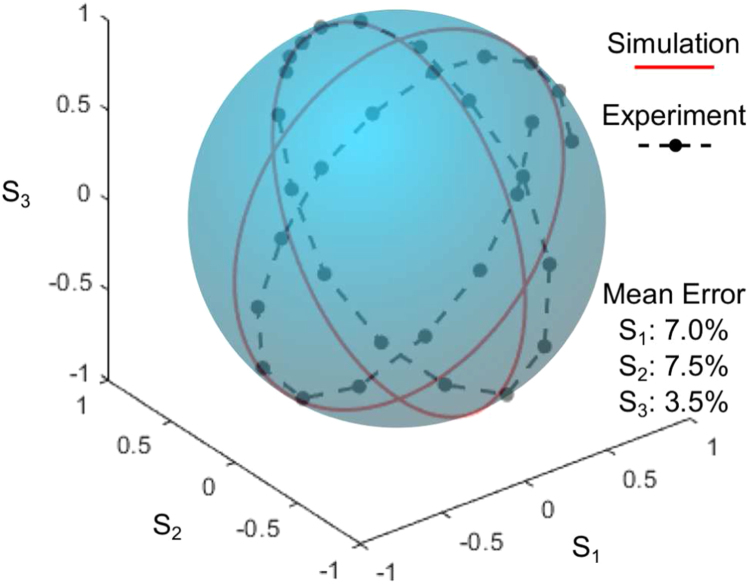
The measured Stokes vector of the beam for an incident horizontally polarized beam by varying the parameters $\phi_{c}$ and $\phi_{d}$. The circle on the plane $S_{1}=0$ represents $\phi_{c}$ in the range [0,2π] and $\phi_{4}=0$. The circle on the plane $S_{2}=0$ represents $\phi_{c}=\pi ∕2$ and $\phi_{d}$ in the range (−0.5π,1.5π). The mean measurement errors of parameters $S_{1},S_{2}$, and $S_{3}$ are 7.0%,7.5%, and 3.5% respectively (normalized by $S_{0}$).

 For the polarization modulation configuration with horizontally polarized input beam, the Stokes vector of the output is ${\left(1\sin\left(\phi_{c}\right)\sin\left(\phi_{d}\right)-\cos\left(\phi_{c}\right)-\sin\left(\phi_{c}\right)\cos\left(\phi_{d}\right)\right)}^{T}$ where $\phi_{c}$ and $\phi_{d}$ are the retardance of the SLM panel during the first and second pass respectively (see supplementary material for the derivation). To better understand the effect of $\phi_{c}$ and $\phi_{d}$ on the output polarization, we plot the Stokes vector by setting $\phi_{d}$ a constant while varying $\phi_{c}$ and vice versa. Fig. 3 is the plot of the Stokes vector by setting $\phi_{c}$ in the range from 0 to 2π with $\phi_{d}=0$ tracing a full circle on the plane $S_{1}=0$. It traces another full circle on the plane $S_{2}=0$ by setting $\phi_{c}=\pi ∕2$ and $\phi_{d}$ in the range from −π∕2 to 3π∕2. The measured Stokes vector using the above settings are also plotted as individual points connected by a dashed line. The mean measurement errors of parameters $S_{1}$, $S_{2}$ and $S_{3}$ are 7.0%,7.5%, and 3.5% respectively (normalized by $S_{0}$). The experimental result matches the theory and traces the same path. It can be seen that the Mueller matrix of the third SLM panel rotates the Stokes vector about the $S_{1}$ axis by an angle of $\phi_{c}$. Meanwhile, the Mueller matrix of the fourth SLM panel rotates the Stokes vector about the $S_{2}$ axis by an angle of $\phi_{d}$.

**Fig. 4:**
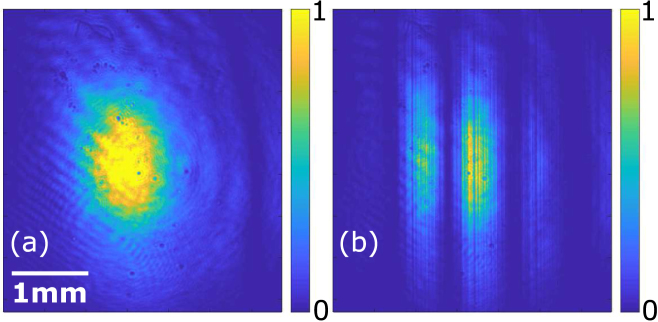
The output beam (a) without (b) with sinusoidal amplitude modulation across the horizontal axis. The scale bar is the same for all subfigures.

**Fig. 5:**
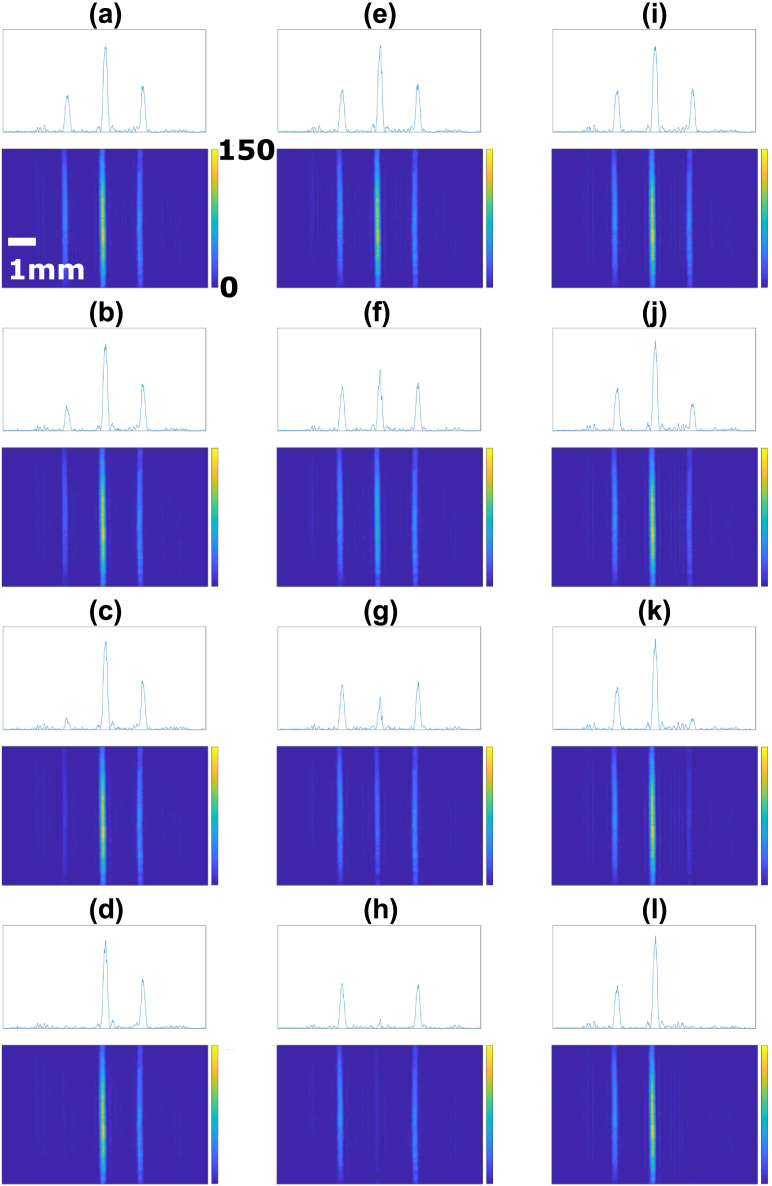
The output beam at the focal plane along with its intensity cross section with SLM phase of a cylindrical lens array of f = 250 mm where three copies of the lens span the beam over 39 SLM pixels, or 3.9 mm. The left focal spot is attenuated by a factor of (a) 0.9, (b) 0.6, (c) 0.3, (d) 0. The middle focal spot is attenuated by a factor of (e) 0.9, (f) 0.6, (g) 0.3, (h) 0. The right focal spot is attenuated by a factor of (i) 0.9, (j) 0.6, (k) 0.3, (l) 0. The scale bar is the same for all subfigures.

Having shown that the 45° degree relative angle between the crystal axis of two SLM panels, mimicked by rotating the polarization by 45°, enable arbitrary polarization modulation. Here we show that the phase and amplitude modulation can be done using identical setup but with incident polarization of 45°, the same as the crystal axis of the first SLM panel. In this configuration, the amplitude of the output beam is attenuated by a factor of $\cos\left(\phi_{d}∕2\right)$ and the phase is increased by an amount of $\phi_{c}+0.5\phi_{d}$. This result is identical to Eq. (7) that describes the proposed VOF-Gen although the derivation is slightly different from Eq. (6). A sinusoidal amplitude modulation is set and the output beam is imaged and shown in Fig. 4(b). Individual pixels of the SLM panel with distinct attenuation can be seen. The output beam without any modulation is displayed in Fig. 4(a). The phase modulation does not affect the intensity distribution or the shape of the beam for a well-aligned system when the output plane is imaged. The imaged output beams with identical amplitude modulation and additional phase modulation are shown in Fig. S2, showing that the additional phase modulation has hardly any noticeable change. To demonstrate simultaneous phase and amplitude modulation, a cylindrical lens array with a focal length of 250 mm is set as SLM phase distribution with individual focal spot attenuated by a certain factor. The beam is imaged at the focal plane, as shown in Fig. 5. Three line-focuses can be seen because three copies of the cylindrical lens over 39 SLM pixels, or 3.9 mm, occupy the spatial extent of the beam. The left focal spot is attenuated by a factor of 0.9, 0.6, 0.3, or 0 (Fig. 5(a)–(d)). The middle focal spot is attenuated by a factor of 0.9, 0.6, 0.3, or 0 (Fig. 5(e)–(h)). The right focal spot is attenuated by a factor of 0.9, 0.6, 0.3, or 0 (Fig. 5(i)–(l)). Different number of line-focuses can be generated by controlling the spatial extend of each cylindrical lens. This is demonstrated in Fig. S3 in the supplementary material.

To evaluate the modulation efficiency of proposed dynamic VOF-Gen (Fig. 1), we remove the quarter-wave-plate and insert a polarizer in double pass configuration (Fig. 2) to mimic the proposed dynamic VOF-Gen in Fig. 1. The power at the laser output and the dynamic VOF-Gen output are measured and the loss of the dynamic VOF-Gen is calculated to be 28±1%. Therefore, the proposed VOF-Gen is expected to have a modulation efficiency of 72±1% with a similar loss. This extrapolation is based on the assumption that the two-dimensional TSLM in the proposed VOF-Gen has the same loss as the CRi SLM we use and the fact that the CRi TSLM is a dual mask TSLM so the laser beam does pass four panels of TSLM in total. Lastly, it is important to note that the proposed dynamic VOF-Gen is flexible with two-dimensional spatial modulation to generate arbitrary VOFs. The TSLM in the proposed dynamic VOF-Gen (Fig. 1) is a two-dimensional dual mask TSLM with crystal axis of the two masks to be 45° with respect to each other.

## 4 Conclusion

We proposed a compact and stable dynamic VOF-Gen that consists of only five optical elements. Arbitrarily structured vector beam can be achieved with a single 2D dual mask transmissive SLM unit and a reflective 4f system. This proposed dynamic VOF-Gen is theoretically 100% efficient. We experimentally demonstrate the proof of principle of the concept with an efficiency of 72%. As a result, this design and demonstration enable dynamic VOF-Gen for a wide range of practical applications, such as focus engineering, optical imaging, laser cutting, optical communication, particle manipulation, and many more.

## CRediT authorship contribution statement

**Billy Lam:** Conceptualization, Methodology, Software, Investigation, Formal analysis, Writing - original draft. **Chunlei Guo:** Conceptualization, Supervision, Writing - review & editing.

## Declaration of Competing Interest

The authors declare that they have no known competing financial interests or personal relationships that could have appeared to influence the work reported in this paper.

## Appendix A Supplementary data

MMC S1 .
