# Supplementary material for: High-efficiency non-diffractive generator of arbitrary vectorial optical fields with minimal optical elements
Source: Opt Commun. 2020 May 15;463:125443. doi: 10.1016/j.optcom.2020.125443 (PMC7097970; doi:10.1016/j.optcom.2020.125443)
Supplement: MMC S1 — . [file mmc1.zip › Supplementary.pdf]

High-efficiency Non-Diffractive Generator of Arbitrary Vectorial Optical Field with Minimal Optical Elements: Supplementary Materials

Billy Lam, Chunlei Guo*

The Institute of Optics, University of Rochester, Rochester, New York, 14627, USA

ARTICLE INFO

Keywords:
Spatial light modulator
Beam shaping
Optical fields

ABSTRACT

This document provides supplementary information to "High-efficiency Vectorial Optical Field Generator with Minimal Optical Elements". This document contains additional data and the detailed derivation of the theory of our design of the vectorial optical field generator (VOF-Gen).

Phase and amplitude modulation

Individual spatial light modulator (SLM) pixel with crystal axis at θ and retardance of ϕ can be written as

$$M(\phi,\theta)=\begin{pmatrix} e^{i\phi}\cos^2\theta+\sin^2\theta & (e^{i\phi}-1)\sin\theta\cos\theta \\ (e^{i\phi}-1)\sin\theta\cos\theta & e^{i\phi}\sin^2\theta+\cos^2\theta \end{pmatrix}. \tag{S1}$$

Here, we will show that the first pass of the dual mask transmissive SLM in the configuration shown in Fig. 1 of the main manuscript will modulate the phase and amplitude of the beam. A horizontally polarized beam, $\mathbf{U}_1(x,y) = (U_1(x,y)\ \ 0)^T$ where T denotes transpose, passing through the first SLM panel with crystal axis at 0° followed by the second SLM panel with crystal axis at 135° followed by a linear polarizer at 0° will result in a field described by

$$\mathbf{U}_2'(x,y)=\begin{pmatrix}1&0\\0&0\end{pmatrix} M(\phi_2,135°)M(\phi_1,0°)\begin{pmatrix}U_1(x,y)\\0\end{pmatrix} \tag{S2}$$

$$=\frac{1}{2}\begin{pmatrix}1&0\\0&0\end{pmatrix}\begin{pmatrix}1+e^{i\phi_2}&1-e^{i\phi_2}\\1-e^{i\phi_2}&1+e^{i\phi_2}\end{pmatrix}\begin{pmatrix}e^{i\phi_1}&0\\0&1\end{pmatrix}\begin{pmatrix}U_1(x,y)\\0\end{pmatrix} \tag{S3}$$

$$=\frac{1}{2}\begin{pmatrix}1+e^{i\phi_2}&1-e^{i\phi_2}\\0&0\end{pmatrix}\begin{pmatrix}e^{i\phi_1}&0\\0&1\end{pmatrix}\begin{pmatrix}U_1(x,y)\\0\end{pmatrix} \tag{S4}$$

$$=\frac{1}{2}\begin{pmatrix}1+e^{i\phi_2}&1-e^{i\phi_2}\\0&0\end{pmatrix}\begin{pmatrix}e^{i\phi_1}U_1(x,y)\\0\end{pmatrix} \tag{S5}$$

$$=\frac{1+e^{i\phi_2}}{2}\begin{pmatrix}e^{i\phi_1}U_1(x,y)\\0\end{pmatrix} \tag{S6}$$

$$=e^{i\frac{\phi_2}{2}}\frac{e^{-i\frac{\phi_2}{2}}+e^{i\frac{\phi_2}{2}}}{2}\begin{pmatrix}e^{i\phi_1}U_1(x,y)\\0\end{pmatrix} \tag{S7}$$

$$=e^{i\frac{\phi_2}{2}}\cos\left(\frac{\phi_2}{2}\right)\begin{pmatrix}e^{i\phi_1}U_1(x,y)\\0\end{pmatrix} \tag{S8}$$

$$=U_1(x,y)e^{i(\phi_1+0.5\phi_2)}\begin{pmatrix}\cos\left(\frac{\phi_2}{2}\right)\\0\end{pmatrix}=\mathbf{U}_2'(x,y) \tag{S9}$$

Upon exiting the linear polarizer, the output beam is attenuated by a factor of $\cos(\phi_2/2)$ and the phase is increased by $\phi_1+0.5\phi_2$. This also occur in the proof of principle device shown in Fig. 2 of the main manuscript. In this configuration, a 45° polarized beam $\mathbf{U}_a(x) = (U_a(x,y)\ \ U_a(x,y))^T/\sqrt{2}$ passes through an SLM panel with crystal axis at 45° followed by a quarter-wave plate (QWP) at 22.5°. Then the beam is reflected in a reflective 4f-system back to the same QWP and SLM at a different transverse position. Lastly, the beam passes through a polarizer. The Jones vector of this output $\mathbf{j}_{mimic}$ is described by

$$\mathbf{j}_{mimic}=\frac{U_a(x,y)}{\sqrt{2}}\begin{pmatrix}1&0\\0&0\end{pmatrix}M(\phi_b,135°)M\left(\frac{\pi}{2},-22.5°\right) \tag{S10}$$

$$\times\begin{pmatrix}1&0\\0&-1\end{pmatrix}M\left(\frac{\pi}{2},22.5°\right)M(\phi_a,45°)\begin{pmatrix}1\\1\end{pmatrix} \tag{S11}$$

$$=U_a(x,y)e^{i(\phi_a+0.5\phi_b)}\begin{pmatrix}\cos\left(\frac{\phi_b}{2}\right)\\0\end{pmatrix} \tag{S12}$$

Polarization modulation

The field is then relay to the second SLM section by a reflective 4f-system. Because the optical fields from the fourier plane to the focus are related by a fourier transform, the optical field at the third SLM panel is

$$\mathbf{U}_3(x,y)=\mathcal{F}\left\{\begin{pmatrix}1&0\\0&-1\end{pmatrix}\mathcal{F}\{\mathbf{U}_2'(x,y)\}\right\} \tag{S13}$$

$$=\mathbf{U}_2'(-x,-y) \tag{S14}$$

A horizontally polarized beam described by

$$\mathbf{U}_3(x,y)=\begin{pmatrix}U_3(x,y)\\0\end{pmatrix} \tag{S15}$$

*Corresponding author
guo@optics.rochester.edu (C. Guo)
ORCID(s): 0000-0001-8525-6301 (C. Guo)

$$= \begin{pmatrix} U_2(-x,-y) \\ 0 \end{pmatrix} \tag{S16}$$

passing through the third SLM panel with crystal axis at 45° followed by the fourth SLM panel with crystal axis at 0° will result in a field described by

$$\mathbf{U}_4'(x,y) = M(\phi_4, 0°)M(\phi_3, 45°)\begin{pmatrix} U_3(x,y) \\ 0 \end{pmatrix} \tag{S17}$$

$$= \frac{1}{2}\begin{pmatrix} e^{i\phi_4} & 0 \\ 0 & 1 \end{pmatrix}\begin{pmatrix} 1+e^{i\phi_3} & -1+e^{i\phi_3} \\ -1+e^{i\phi_3} & 1+e^{i\phi_3} \end{pmatrix}\begin{pmatrix} U_3(x,y) \\ 0 \end{pmatrix} \tag{S18}$$

$$= \frac{1}{2}\begin{pmatrix} e^{i\phi_4} & 0 \\ 0 & 1 \end{pmatrix}\begin{pmatrix} (1+e^{i\phi_3})U_3(x,y) \\ (-1+e^{i\phi_3})U_3(x,y) \end{pmatrix} \tag{S19}$$

$$= \frac{1}{2}\begin{pmatrix} e^{i\phi_4}(1+e^{i\phi_3})U_3(x,y) \\ (-1+e^{i\phi_3})U_3(x,y) \end{pmatrix} \tag{S20}$$

$$= \frac{1}{2}e^{i\frac{\phi_3}{2}}\begin{pmatrix} e^{i\phi_4}\left(e^{-i\frac{\phi_3}{2}} + e^{i\frac{\phi_3}{2}}\right)U_3(x,y) \\ \left(-e^{-i\frac{\phi_3}{2}} + e^{i\frac{\phi_3}{2}}\right)U_3(x,y) \end{pmatrix} \tag{S21}$$

$$= e^{i\frac{\phi_3}{2}}\begin{pmatrix} e^{i\phi_4}\cos\left(\frac{\phi_3}{2}\right)U_3(x,y) \\ i\sin\left(\frac{\phi_3}{2}\right)U_3(x,y) \end{pmatrix} \tag{S22}$$

$$= e^{i(\phi_3/2+\phi_4)}\begin{pmatrix} \cos\left(\frac{\phi_3}{2}\right)U_3(x,y) \\ \sin\left(\frac{\phi_3}{2}\right)U_3(x,y)e^{-i\left(\phi_4-\frac{\pi}{2}\right)} \end{pmatrix} \tag{S23}$$

The field at the third SLM panel is the invert of that at the second SLM panel by Eq. (S16), the expression becomes

$$\mathbf{U}_4'(x,y) = e^{i(\phi_3/2+\phi_4)}U_2'(-x,-y)\begin{pmatrix} \cos\left(\frac{\phi_3}{2}\right) \\ \sin\left(\frac{\phi_3}{2}\right)e^{-i\left(\phi_4-\frac{\pi}{2}\right)} \end{pmatrix} \tag{S24}$$

Combining Eq. (S9), the final expression becomes

$$\mathbf{U}_4' = U_1(-x,-y)\cos\left(\frac{\phi_2}{2}\right)e^{i(\phi_1+0.5\phi_2+0.5\phi_3+\phi_4)} \times \begin{pmatrix} \cos\left(\frac{\phi_3}{2}\right) \\ \sin\left(\frac{\phi_3}{2}\right)e^{-i\left(\phi_4-\frac{\pi}{2}\right)} \end{pmatrix} \tag{S25}$$

which is the same as Eq. (13) in the main manuscript.

0.1. Mueller Calculus

The Poincaré sphere is a three-dimensional representation of the polarization state. The parameters that describe the polarization state are called the Stokes parameters, which can be written in a vector form as $\mathbf{S} = (S_0\ \ S_1\ \ S_2\ \ S_3)^T$ where T is the transpose. The Stokes parameters are defined by

$$S_0 =< E_H >^2 + < E_V >^2 \tag{S26}$$

$$S_1 =< E_H >^2 - < E_V >^2 \tag{S27}$$

$$S_2 =< E_{45} >^2 - < E_{135} >^2 \tag{S28}$$

$$S_3 =< E_l >^2 - < E_r >^2 \tag{S29}$$

where the subscripts refers to the three different orthogonal bases of polarization state: the standard Cartesian basis $(\hat{H}, \hat{V})$, a Cartesian basis rotated by 45° $((\hat{H}+\hat{V})/\sqrt{2}, (\hat{H}-\hat{V})/\sqrt{2})$, and a circular basis $(\hat{l}, \hat{r})$. The Mueller matrix is used to describe the transformation of the polarization state as the beam propagates through an optical element. The Mueller matrix of an SLM pixel with crystal axis θ and retardance ϕ can be written as

$$\mathbf{M}(\phi,\theta) = \mathbf{R}(\theta)\begin{pmatrix} 1 & 0 & 0 & 0 \\ 0 & 1 & 0 & 0 \\ 0 & 0 & \cos\phi & -\sin\phi \\ 0 & 0 & \sin\phi & \cos\phi \end{pmatrix}\mathbf{R}(-\theta) \tag{S30}$$

where

$$\mathbf{R}(\theta) = \begin{pmatrix} 1 & 0 & 0 & 0 \\ 0 & \cos 2\theta & -\sin 2\theta & 0 \\ 0 & \sin 2\theta & \cos 2\theta & 0 \\ 0 & 0 & 0 & 1 \end{pmatrix} \tag{S31}$$

is the rotation matrix that rotates the local frame to the laboratory frame. The retardance ϕ for an SLM is controllable. Therefore The SLM introduce a variable rotation on the Poincaré sphere about a fixed rotation axis defined by the orientation of the crystal axis of the SLM panel.

In order to achieve arbitrary polarization state, two linearly independent rotation about two distinct axes is required. Two SLM pixels in series whose crystal axis are 45° with respect to each other would constitute as two rotations about two orthogonal rotation axis, which is an effective method to control the polarization state. The Mueller matrix of the third SLM panel $\mathbf{M}(\phi_3, 45°)$ rotates the Stokes vector about S_2 axis by an angle of ϕ_3. Meanwhile, the Mueller matrix of the fourth SLM panel $\mathbf{M}(\phi_4, 0°)$ rotates the Stokes vector about the S_1 axis by an angle of ϕ_4. This effect is depicted in Fig. S1, the plot of the Stokes vector by setting ϕ_3 in the range from 0 to 2π with $\phi_4 = 0$ tracing a full circle. It also trace another semicircle by setting $\phi_3 = \pi/2$ and ϕ_4 in the range from 0 to π. The Stokes vector representing the output beam (Eq. (13) is $(1\ \ \cos\phi_3\ \ \sin\phi_3\sin\phi_4\ \ -\sin\phi_3\cos\phi_4)^T$, which also shows the effect of ϕ_3 and ϕ_4 as two orthogonal rotations described above. The detailed derivation of the Stokes vector can be found in the next section.

Although the Poincaré sphere does not contain information about the phase, it is a great visualization of the evolution of the polarization state.

Two SLM pixels in series whose crystal axis are 45° with respect to each other can also be utilized for phase and amplitude modulation. The phase modulation by using an incident

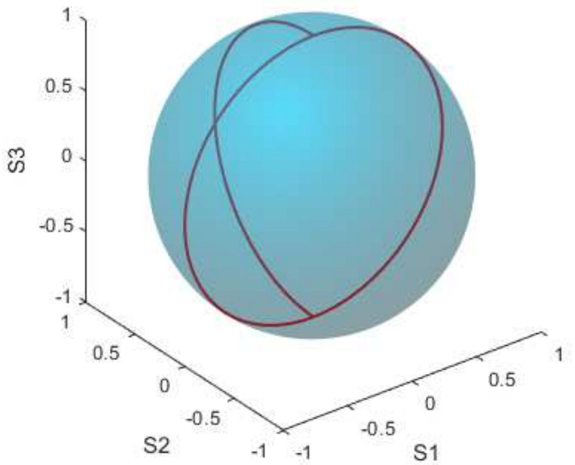

Figure S1: The final Stokes vector of the beam for an incident horizontally polarized beam by varying the parameters ϕ_3 and ϕ_4. The full circle represents ϕ_3 in the range $[0, 2\pi]$ and $\phi_4 = 0$. The semicircle represents $\phi_3 = \pi/2$ and ϕ_4 in the range $[0, \pi]$.

polarization of horizontal and crystal axis of 0° would be a rotation about the S_1 axis, so the polarization is unchanged. The SLM pixel with 45° crystal axis rotates the state of polarization about the S_2 axis. The initial polarization state can be rotated to the opposite pole, the orthogonal polarization state, by a 180° rotation. Thus, a polarizer can be added for variable attenuation with maximum modulation.

Dual mask transmissive SLM usually has their crystal axis 90° with respect to each other for phase and amplitude modulation. This leads to rotation of the Stokes vector about the same rotation axis but opposite angle. Such configuration is less versatile compared to crystal axes being 45° with respect to each other.

Polarization modulation (Mueller Calculus)

The underlying behavior of the Stokes vector as the beam propagates through the third and fourth SLM panels of the proposed VOF-Gen is briefly explained in the manuscript. The Mueller matrix of the third SLM and fourth SLM panels rotates the Stokes vector about two orthogonal axis. A brief derivation is provided below.

The Mueller matrix of a SLM pixel with crystal axis θ and retardance ϕ can be written as

$$\mathbf{M}(\phi,\theta) = \mathbf{R}(\theta)\begin{pmatrix} 1 & 0 & 0 & 0 \\ 0 & 1 & 0 & 0 \\ 0 & 0 & \cos\phi & -\sin\phi \\ 0 & 0 & \sin\phi & \cos\phi \end{pmatrix}\mathbf{R}(-\theta) \quad \text{(S32)}$$

where

$$\mathbf{R}(\theta) = \begin{pmatrix} 1 & 0 & 0 & 0 \\ 0 & \cos 2\theta & -\sin 2\theta & 0 \\ 0 & \sin 2\theta & \cos 2\theta & 0 \\ 0 & 0 & 0 & 1 \end{pmatrix} \quad \text{(S33)}$$

is the rotation matrix that rotate the local frame to the laboratory frame. When the incident horizontally polarized beam propagate through the third and fourth SLM panel, the Stokes vector becomes

$$\mathbf{S}_{final} = \mathbf{M}(\phi_4, 0°)\mathbf{M}(\phi_3, 45°)\begin{pmatrix} 1 \\ 1 \\ 0 \\ 0 \end{pmatrix} \quad \text{(S34)}$$

Plugging Eq. (S32, S33) into Eq. (S34) yields

$$\mathbf{S}_{final} = \begin{pmatrix} 1 \\ \cos\phi_3 \\ \sin\phi_3 \sin\phi_4 \\ -\sin\phi_3\cos\phi_4 \end{pmatrix} \quad \text{(S35)}$$

It is unclear from this expression what effects ϕ_3 and ϕ_4 has on the Stokes vector. However, the behavior becomes evident once the Stokes vector is plotted by setting ϕ_4 a constant while varying ϕ_3 and vice versa. Fig. S1 is the plot of the Stokes vector by setting ϕ_3 in the range from 0 to 2π with $\phi_4 = 0$ tracing a full circle. It also trace another semicircle by setting $\phi_3 = \pi/2$ and ϕ_4 in the range from 0 to π. It can be seen that the Mueller matrix of the third SLM panel rotates the Stokes vector about the S_1 axis by an angle of ϕ_3. Meanwhile, the Mueller matrix of the fourth SLM panel rotates the Stokes vector about the S_1 axis by an angle of ϕ_4.

The Mueller matrix of the third SLM and fourth SLM panels rotates the Stokes vector about two orthogonal axis because the crystal axis is 45° with respect to each other. We mimic this effect by rotating the polarization by 45° while using the same SLM panel. The horizontally polarized beam first passes through the SLM panel with crystal axis at 45°. The field is then relay to the same SLM panel while double passing a quarter wave plate, which act as a half-wave plate and rotate the polarization by 45°. The Stokes vector of the beam becomes

$$\mathbf{S}_{mimic} = \mathbf{M}(\phi_d, -\frac{\pi}{4})\mathbf{M}\left(\frac{\pi}{2}, -\frac{\pi}{8}\right)\mathcal{R}\mathbf{M}\left(\frac{\pi}{2}, \frac{\pi}{8}\right)\mathbf{M}(\phi_c, \frac{\pi}{4})\begin{pmatrix} 1 \\ 1 \\ 0 \\ 0 \end{pmatrix} \quad \text{(S36)}$$

where

$$\mathcal{R} = \begin{pmatrix} 1 & 0 & 0 & 0 \\ 0 & 1 & 0 & 0 \\ 0 & 0 & -1 & 0 \\ 0 & 0 & 0 & -1 \end{pmatrix} \quad \text{(S37)}$$

is the reflection matrix of the mirror. Plugging Eq. (S32, S33) into Eq. (S36) yields

$$\mathbf{S}_{mimic} = \begin{pmatrix} 1 \\ \sin(\phi_c)\sin(\phi_d) \\ -\cos(\phi_c) \\ -\sin(\phi_c)\cos(\phi_d) \end{pmatrix} \quad \text{(S38)}$$

To better understand the effect of ϕ_c and ϕ_d, we plot the Stokes vector by setting ϕ_d a constant while varying ϕ_c and vice versa. Fig. 3 in the main manuscript is the plot of the Stokes vector by setting ϕ_c in the range from 0 to 2π with $\phi_d = 0$ tracing a full circle on the plane $S_1 = 0$. It also trace a fullcircle on the plane $S_2 = 0$ by setting $\phi_c = \pi/2$ and ϕ_d in the range from 0 to 2π. It can be seen that the Mueller matrix of the third SLM panel rotates the Stokes vector about the S_1 axis by an angle of ϕ_c. Meanwhile, the Mueller matrix of the fourth SLM panel rotates the Stokes vector about the S_2 axis by an angle of ϕ_d.

Corresponding SLM pixels of the two sections

As seen in Eq. (S14), the field is inverted by the reflective 4f-system to pass the SLM the second time. Hence, the TSLM should be divided into left and right half for the two sections of modulation given that the horizontal axis has more pixels. This means that the 4f-system is aligned exactly symmetric about the center pixel of the SLM. Naming the pixels as (x_i, y_j) where $i \in \{1, 2, ..., N_x\}$ and $j \in \{1, 2, ..., N_y\}$ with N_x and N_y being the number of pixels along that axis. The corresponding pixel between the first and second section of modulation is related by $(x_i, y_j) \sim (x_{N_x-i+1}, y_{N_y-j+1})$. By Eq. (S9, S25), the four degrees of freedoms are tailored as follows:

$$\text{Retardance} = -\phi_4(x_{N_x-i+1}, y_{N_y-j+1}) + \frac{\pi}{2} \tag{S39}$$

$$\text{Polarization ratio} = \left| \cot\left(\frac{\phi_3(x_{N_x-i+1}, y_{N_y-j+1})}{2} \right) \right| \tag{S40}$$

$$\text{Amplitude} = U_1(x_i, y_i) \cos\left(\frac{\phi_2(x_i, y_j)}{2} \right) \tag{S41}$$

$$\begin{aligned} \text{Phase} = {} & \phi_1(x_i, y_j) + 0.5\phi_2(x_i, y_j) \\ & + 0.5\phi_3(x_{N_x-i+1}, y_{N_y-j+1}) \\ & + \phi_4(x_{N_x-i+1}, y_{N_y-j+1}) \end{aligned} \tag{S42}$$

Additional data for phase and amplitude modulation

Using the setup shown in Fig. 3 of the main manuscript with incident polarization of 45°, simultaneous phase and amplitude modulation can be achieved. A linear amplitude modulation is set and the output beam is imaged and shown in Fig. S2(b). Individual pixel of the SLM panel with distinct attenuation can be seen. The output beam without any modulation is displayed in Fig. S2(a). The identical amplitude modulation with additional phase modulation are shown in Fig. S2(c) and S2(d). The output beam with phase modulation (Fig. S2(c)) is near identical to that without any modulation (Fig. S2(a)). Similarly, the output beam with both

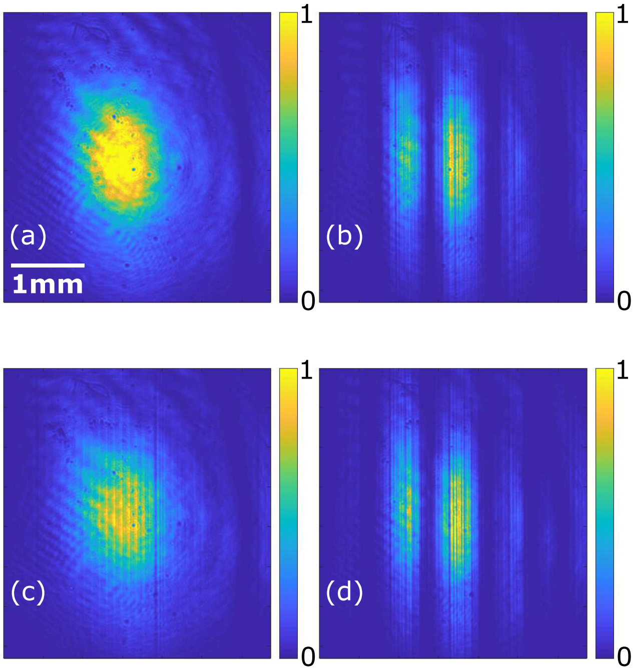

Figure S2: The output beam (a) without (b) with sinusoidal amplitude modulation (c) with phase modulation (b) with both phase and amplitude modulation across the horizontal axis.

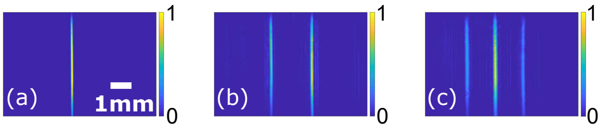

Figure S3: The output beam at the focal plane with SLM phase of (a) a cylindrical lens of $f = 250$ mm (b) cylindrical lens array where two copies of the lens span the beam over 38 SLM pixels, or 3.8 mm. (c) cylindrical lens array where three copies of the lens span the beam over 39 SLM pixels. The scale bar is the same for all figures.

phase and amplitude modulation (Fig. S2(d)) is near identical to that with amplitude modulation (Fig. S2(b)). The phase modulation has hardly any noticeable effect on the intensity distribution or the shape of the beam.

To demonstrate the phase modulation, a cylindrical lens array with focal length of 250 mm is inputted into to the SLM and the beam is imaged at the focal plane, shown in Fig. S3. Multiple line-focuses can be seen at the focal plane depending on the spatial extent of the cylindrical lens. There is a single line-focus when the lens spans over 41 SLM pixels, or 4.1 mm (Fig. S3(a)). Similarly, there are 2 (3) line-focuses when the lens span over 19 (13) SLM pixels (see Fig. S3(b)-(c)). Amplitude modulation can also be added to this cylindrical lens array. Figure 5 in the main manuscript demonstrates the simultaneous phase and amplitude modulation by attenuating individual focal spot of the cylindrical lens array.
